# Severe potential drug-drug interactions in older adults with dementia and associated factors

**DOI:** 10.6061/clinics/2016(01)04

**Published:** 2016-01

**Authors:** Michele Bogetti-Salazar, Cesar González-González, Teresa Juárez-Cedillo, Sergio Sánchez-García, Oscar Rosas-Carrasco

**Affiliations:** I Hospital Angeles Mocel, Department of Internal Medicine, Mexico City/DF, Mexico; II National Geriatrics Institute, Mexico City/DF, Mexico; III Epidemiologic and Health Service Research Unit, Aging Area. Mexican Institute of Social Security, and Faculty of High Studies (FES) Zaragoza. National Autonomous University of Mexico, Mexico City/DF, Mexico; IV Epidemiologic and Health Service Research Unit, Aging Area. Mexican Institute of Social Security, Mexico City/DF, Mexico

**Keywords:** Potential Drug-Drug Interactions, Older People, Dementia

## Abstract

**OBJECTIVE:**

To identify the main severe potential drug-drug interactions in older adults with dementia and to examine the factors associated with these interactions.

**METHOD:**

This was a cross-sectional study. The enrolled patients were selected from six geriatrics clinics of tertiary care hospitals across Mexico City. The patients had received a clinical diagnosis of dementia based on the current standards and were further divided into the following two groups: those with severe drug-drug interactions (contraindicated/severe) (n=64) and those with non-severe drug-drug interactions (moderate/minor/absent) (n=117). Additional socio-demographic, clinical and caregiver data were included.

Potential drug-drug interactions were identified using Micromedex Drug Reax 2.0^®^ database.

**RESULTS:**

A total of 181 patients were enrolled, including 57 men (31.5%) and 124 women (68.5%) with a mean age of 80.11±8.28 years. One hundred and seven (59.1%) patients in our population had potential drug-drug interactions, of which 64 (59.81%) were severe/contraindicated. The main severe potential drug-drug interactions were caused by the combinations citalopram/anti-platelet (11.6%), clopidogrel/omeprazole (6.1%), and clopidogrel/aspirin (5.5%). Depression, the use of a higher number of medications, dementia severity and caregiver burden were the most significant factors associated with severe potential drug-drug interactions.

**CONCLUSIONS:**

Older people with dementia experience many severe potential drug-drug interactions. Anti-depressants, antiplatelets, anti-psychotics and omeprazole were the drugs most commonly involved in these interactions. Despite their frequent use, anti-dementia drugs were not involved in severe potential drug-drug interactions. The number and type of medications taken, dementia severity and depression in patients in addition to caregiver burden should be considered to avoid possible drug interactions in this population.

## INTRODUCTION

Anatomical and physiological changes and impairments in multiple organs and systems associated with aging can affect the pharmacokinetics and pharmacodynamics of drugs 1. Elderly patients with dementia are a group of great interest in the study of potential drug-drug interactions (DDIs). In addition to decreased hepatic metabolism in older adults, the presence of cognitive deficits or neuropsychiatric symptoms (e.g., mood, behavioral and perception disorders) may prompt the prescription of a wide variety of drugs, including anti-psychotics, antidepressants, anxiolytics, mood stabilizers and anti-dementia drugs 2-4. Most of these drugs are extensively metabolized in the liver by cytochrome p450 isoenzymes (CYP1A2, CYP2C19, CYP2D6, CYP3A4, CYP2D6, CYP3A4, CYP1A2, CYP2D6, CYP3A4, CYP3A4, and others) 5,6.

A high associated comorbidity has been previously reported for these patients, of whom 61% have three or more diseases. Of them, musculoskeletal, cardiovascular, metabolic/endocrine and gastrointestinal disorders are the most common 7-9. Many of the medications prescribed to treat these disorders are metabolized in the liver by cytochrome p450 isoenzymes</underline> similar to the aforementioned psychotropics, including antiplatelet drugs (clopidogrel and aspirin), non-steroidal anti-inflammatory drugs, omeprazole, antidiabetic agents (metformin) and others, resulting in an increase in poor quality prescription, potential DDIs and adverse reactions.

Over time, new drugs (psychotropic and anti-dementia drugs) will emerge for the treatment of dementia patients, giving rise to new interactions and challenges for clinicians.

The factors associated with potential DDIs in elderly patients without dementia have been previously reported and include age, female gender, polypharmacy and high comorbidity 10-13. Limited research has been performed to date on potential DDIs in patients with dementia as well as the associated factors. Moreover, the findings of previous studies have been controversial; some studies have reported a low frequency of potential DDIs in patients with dementia compared to those without dementia 2,3,14, and others have examined specific drug types, focusing primarily on medications with anticholinergic properties 15.

To our knowledge, there are limited data available on the factors contributing to severe potential DDIs caused by drugs prescribed to dementia patients. Thus, it is important to identify the main severe potential DDIs and the factors associated with these interactions in older adults with dementia.

## METHODS

We used the database from the “Validation of the Quality of Life in Alzheimer's Disease (QOL-AD) scale in Mexican patients with dementia” 16, a cross sectional study the quality of life of dementia patients. The study involved both primary caregivers and outpatients with dementia from different health institutions located in Mexico City. The patients were interviewed at scheduled appointments at six general hospitals. The study took place from January 2007 to January 2010 and included patients aged 60 years or older with dementia. The enrolled patients were required to be able to read and write. The caregiver was included if he/she was able to read and write, was not paid and generally knew more than the patient about the his or her environment and care. The main exclusion criteria were as follows: (i) the presence of acute and/or exacerbated chronic disease within the 30 days before the interview that could have affected the quality of the responses to the questionnaires, as determined by the study's medical staff; (ii) decreased alertness (for any reason); (iii) severe aphasia; (iv) visual and hearing impairments, resulting in difficulty for the patient/caregiver with filling out the questionnaires; (v) the presence of other neurological diseases that could have influenced the diagnosis of dementia; and (vi) institutionalization of the patient.

### Patient variables

We included data from 181 outpatients who were 60 years of age or older and had a clinical diagnosis of dementia. Dementia (e.g., Alzheimer's, vascular, mixed, frontotemporal and Lewy body dementia) had been previously diagnosed by a group of geriatricians dedicated to the study of dementia according to international criteria; these criteria have been described in a previous publication 17.

### Measurements

#### Interaction analysis

Potential DDIs were identified using Micromedex Drug Reax 2.0® database. We focused our study on the worst potential DDIs because they tend to have a greater clinical impact on patients' well-being. For this analysis, potential DDIs were classified into the following 2 general categories: contraindicated/severe potential DDIs were combined in a “severe interactions” category to represent the worst possible outcomes of a given drug combination and moderate/minor/absent potential DDIs were combined to form a “non-severe interaction” group. The groups were labeled and compared as “severe” *versus* “non-severe”.

### Number of medications

A list of medications taken by the elderly subjects was generated based on a review of the clinical records and it was confirmed by the physicians. The quantitative variable in this study was the total number of medications taken.

### Comorbidity

We used a Spanish version of the Charlson index. This scale includes a list of 19 diseases and their complications. Comorbidities were determined by caregiver interviews and a review of the clinic's records and the total score was used for this variable 18.

Caregiver burden was determined by the Screen for Caregiver Burden (25-item scale). It has been adapted and validated for use in the Mexican population. We analyzed the total score achieved on this test (ranging from 0 to 100) 19.

Another measure employed was a Spanish version of the Dysexecutive Questionnaire (DEX), a 20-item questionnaire that uses a Likert scale ranging from 0 to 4 points. For this measure, we assessed the total score (ranging from 0 to 80), with the primary caregiver as the interviewed party (proxy) 20.

The Mini-Mental State Examination (MMSE) was used as an indirect indicator of dementia severity. We used a version that has been previously validated for use in the Mexican population 21.

The Barthel index was applied to assess the Spanish version of the Basic Activities of Daily Living (ADL) scale. We analyzed the total score (ranging from 0 to 100) 22.

The Lawton Scale was used to evaluate instrumental activities of daily living (IADL). This scale contains eight items, and we analyzed the cumulative score (ranging from 0 to 8) 23.

To evaluate the presence of depression, we used the depression subscale of the Neuropsychiatric Inventory (NPI-D). The frequency score was classified as presence=1 or absence=0 for a 12-item inventory validated for use in the Mexican population that, was completed by the caregivers 24.

The original project was approved by the National Scientific Research Committee of IMSS (Approval Number 2006-785-065). All patients and caregivers provided written informed consent.

Descriptive analyses were performed using frequencies, percentages and Pearson's Chi^2^ test (comparative analysis) for qualitative variables. The mean ± SD and Student's t-test (comparative analysis) were used to analyze quantitative variables. The variables associated with potential DDIs (*p*<0.05) were included in a multivariate model using multiple logistic regression. We used STATA® version 11 software (StataCorp, 2009) to perform analysis.

## RESULTS

We evaluated 181 outpatients from the original study, including 57 males (31.5%) and 124 females (68.5%) with a mean age of 80.11±8.28 years. The patient characteristics are displayed in Table 1. The prevalence of potential DDIs in our population was 59.1%, with 59.8% being classified as severe. Anti-dementia drugs were the most widely used medications (58.6%), followed by anti-depressants (44.2%), anti-psychotics (25.4%) and, to a much lesser degree, sedatives (12.2%). However, none of the anti-dementia drugs were classified as “severe DDIs” (Tables 1 and 2).

The top ten potential DDIs are included in Table 2. The most prevalent interaction in our population occurred with the combined use of citalopram and antiplatelet drugs (n=21; 11.6%) (Table 2).

The independent variables were compared according to the severity of the potential DDIs (severe or non-severe). Comorbidity, depression, polypharmacy, dementia severity and caregiver burden were the most significant factors and were thus used in comparative analysis (Table 3).

The significant variables were included in the final regression model. This model revealed that the variables associated with severe potential DDIs were depression, the number of drugs taken, dementia severity and caregiver burden. Depression was by far the most significant, followed by the number of drugs taken. In addition, caregiver burden and dementia severity exhibited positive but diminished associations with the severe potential DDIs (Table 4).

## DISCUSSION

This study provides insights into the frequency of severe potential DDIs in older people with dementia (35.3%). The frequency observed in this study is higher than that of a previous report (26.6%) 25. We speculate that this difference between studies could be due to the higher frequency of neuropsychiatric symptoms in this study (depression was the only factor associated with severe interactions). Other studies have found a similar high frequency of the use of antidepressants, anxiolytics and antipsychotics in patients with dementia 2,15,25,26.

One of the main objectives of this study was to identify the main severe DDIs because they are the most likely to have poor outcomes in the clinical setting; however, the results of studies based on Micromedex reports should be considered with caution. For example, the main potential DDI (Table 2) determined in this study (citalopram/antiplatelet) according to Micromedex database has been shown to have null or discrete associations with the risk of gastrointestinal bleeding in several clinical contexts in previous clinical studies. Therefore, further studies should be performed to determine the real risk of bleeding 27,28.

Conflicting results have been reported regarding the increased risk of the second most frequent potential DDI observed in this study (clopidogrel/omeprazole). Studies based on Micromedex reports have indicated that their concurrent use may result in an increased risk of bleeding. However, a recent clinical study found that in healthy volunteers omeprazole, esomeprazole and lansoprazole caused a relative decrease in exposure to the active metabolite of clopidogrel by of up to 50%, leading to modest decreases in its antiplatelet effect 29. Also in contrast to data from Micromedex, epidemiological studies have provided no evidence that this drug combination increases the risk of gastrointestinal bleeding, including patients who are receiving aspirin and clopidogrel. Even in patients receiving aspirin and clopidogrel, prophylactic use of a Proton Pump Inhibitors (PPI) has been shown to reduce the rate of upper gastrointestinal bleeding 30. Similar findings have been reported regarding a potential DDI between clopidogrel and paroxetine; while numerous studies have indicated a high risk of bleeding, this result has essentially been based on the evaluation of registries and retrospective or case–control studies 31. A recent basic sciences study of healthy volunteers has found that paroxetine inhibits formation of the active metabolite of clopidogrel, thereby modifying the pharmacodynamics of this product in terms of its antiplatelet effects and consequently, its efficacy 32.

Regarding the factors associated with severe potential DDIs, the final regression model and comparative analysis revealed that the number of medications taken is a risk factor for the presence of severe potential DDIs, in agreement with other studies analyzing these interactions in older adults with and without dementia 2,10,11,9,33.

A secondary objective of this study was to identify different factors associated with the presence of potential DDIs; in this regard, depression was the main neuropsychiatric symptom associated with severe potential DDIs. One of the most widely used treatments for depression is the selective serotonin reuptake inhibitors (SSRIs). SSRIs are currently recommended as the first-line treatment for depression in older adults according to several clinical guidelines 34,35. This group of antidepressant should be used with caution in these patients. Clinical studies should be performed to confirm these findings.

A modest association was found between caregiver burden and severe potential DDIs (Tables 3 and 4). Previous reports have shown that caregivers tend to have greater physical and psychological burdens than the general population, and these burdens have been demonstrated to result in hospitalization of the patients who they care for 36. Caregivers may play an important role in drug administration, and when they are well trained, their help is very important 36. However, in a situation in which dementia represents a heavy burden, a caregiver's persistence could cause physicians to prescribe more or specific medications to control a patient's neuropsychiatric symptoms, leading to severe potential DDIs and resulting in supplementary non-pharmacological treatment.

A greater number of diseases affected the patients with a lower MMSE score than those with a higher MMSE score; therefore, the increased numbers of prescription drugs taken by the patients in this study may be explained by the successive selection of patients attending memory clinics and may not be directly related to their cognitive statuses. Therefore, longitudinal studies should be conducted to confirm this relationship.

There were several limitations of this study. It is important to note that the hospitals at which the outpatients were recruited are specialized institutions, and this study did not include institutionalized or community-dwelling older adults. Thus, our results can only be applied to institutions with similar characteristics. In addition, this was a cross-sectional study, and further research examining the longitudinal association between caregiver burden and other factors and severe potential DDIs is necessary.

Another limitation was the lack of data on the outcomes of the potential DDIs identified, which is attributed to the nature of the study. The scores of the MMSE, which was used as a measure of the severity of dementia, should be considered with caution due to the limitations associated with its use in older adults with a low education level.

Despite these limitations, we believe that this study contributes to current knowledge of the frequency of potential DDIs and the relationships between severe potential DDIs and the contrubuting factors in dementia patients.

## AUTHOR CONTRIBUTIONS

Bogetti-Salazar M assisted with the data collection, protocol development and manuscript preparation. González-González C, Juárez-Cedillo T and Sánchez-García S contributed to the protocol development and manuscript preparation. Rosas-Carrasco O contributed to the data collection, protocol development, statistical analysis and manuscript preparation.

## Figures and Tables

**Table 1:** General patient characteristics.

Factors	N=181 (%)
Age	80.11±8.28
Sex	
Male	57 (31.50)
Female	124 (68.50)
Schooling (years)	7.62±5.34
Total comorbidity score (Charlson index)	2.56±1.57
Number of drugs	5.20±3.04
Executive function (DEX) total score	17.54±18.38
Dementia severity (MMSE) total score	16.80±6.70
ADL (Barthel)	73.92±29.51
IADL (Lawton)	5.0±5.33
Caregiver burden (SCB)	21.91±15.97
NPI Depression item (yes)	111 (61.33)
Drug-drug interactions	
Yes	107 (59.10)
No	74 (40.90)
Severity of Interactions	
Mild	3 (2.80)
Moderate	40 (37.38)
Severe/contraindicated	64 (59.81)

Mini-Mental State Examination (MMSE); Dysexecutive Questionnaire (DEX); Barthel Activities of Daily Living scale (ADL); Lawton Instrumental Activities Daily Living scale (IADL); 12-item Neuropsychiatric Inventory (NPI-D); Screen for Caregiver Burden (SCB).

**Table 2:** Top ten severe potential DDIs (severe and contraindicated).

Type	Effect	n	Interaction (%)	Patient (%)
Citalopram/ Anti-platelet	Concurrent use may result in an increased risk of bleeding. (Documentation: Good)	21	6.7	11.6
Clopidogrel/ Omeprazole	Concurrent use may result in reduction in clinical efficacy of clopidogrel and increased risk of thrombosis. (Documentation: Excellent)	11	3.5	6.1
Clopidogrel/ Aspirin	Concurrent use may result in an increased risk of bleeding. (Documentation: Fair)	10	3.2	5.5
Citalopram/ Omeprazole	Concurrent use may result in increased citalopram exposure and risk of QT interval prolongation. (Documentation: Fair)	8	2.5	4.4
Escitalopram/ Anti-platelet	Concurrent use may result in an increased risk of bleeding. (Documentation: Good)	5	1.6	2.7
Citalopram/ Quetiapine	Concurrent use may result in increased risk of QT interval prolongation. (Evidence level: Fair)	5	1.6	2.7
Paroxetine/ Anti-platelet	Concurrent use may result in an increased risk of bleeding. (Documentation: Good)	4	1.3	2.2
Amlodipine/ Clopidogrel	Concurrent use may result in decreased antiplatelet effects and increased risk of thrombotic events. (Documentation: Excellent)	3	0.3	1.6
Citalopram/ Risperidone	Concurrent use may result in increased risks of QT interval prolongation and torsade de pointes. (Documentation: Good)	3	0.3	1.6
Citalopram/ Haloperidol	Concurrent use may result in an increased risk of QT interval prolongation. (Documentation: Good)	3	0.3	1.6

Documentation: Excellent (controlled studies have clearly established the existence of the interaction), Good (documentation strongly suggests that the interaction exists, but well-controlled studies are lacking), Fair (available documentation is poor, but pharmacologic considerations have led clinicians to suspect that the interaction exists; or the documentation is good for a pharmacologically similar drug), and Unknown (Unknown). The documentation is based on information obtained from Micromedex^®^.

**Table 3:** Comparative analysis of the relationships between potential severe (contraindicated/severe) and non-severe (moderate/mild/none) interactions and other factors.

Factors	Severe interactions Mean ± SD	Non-severe interactions Mean ± SD	*p*-value
Age (years)	80.89±7.6	79.6±8.5	0.35
Sex			
Female n (%)	83 (70.94)	41 (64.0)	0.34
Schooling	8.7±5.5	7.0±5.1	0.05
Comorbidity (Charlson index)	3.0±1.7	2.2±1.4	0.002
Number of drugs	7.68±2.6	3.8±2.2	<0.001
Executive function (DEX)	28.1±28.6	27.2±18.8	0.75
Dementia severity (MMSE)	18.6±5.9	15.7±6.8	0.005
Activities of daily living (Barthel index)	70.9±30.1	75.5±29.1	0.31
Instrumental activities (Lawton)	4.2±4.8	5.43±5.5	0.14
Caregiver burden (SCB 25-item)	25.3±18.6	20.0±14.0	0.03
Depression (NPI) (yes) n (%)	48 (75.0)	63 (53.85)	0.005

Pearson's Chi^2^ test was used for qualitative variables.

Student's t-test was used for quantitative variables.

Mini-Mental State Examination (MMSE), Screen for Caregiver Burden (SCB), Neuropsychiatric Inventory (NPI).

**Table 4:** Factors associated with the presence of severe (contraindicated/severe) potential drug-drug interactions.

Variables	OR	CI (95%)	*p-*value
Dementia severity (MMSE) total score	1.08	1.01-1.16	0.01
Comorbidity (Charlson index) total score	0.89	0.64-1.20	0.47
Number of medications	1.88	1.53-2.32	<0.001
Caregiver burden (SCB) total score	1.03	1.00-1.06	0.01
Depression (NPI) (yes)	3.19	1.24-8.20	0.01

Log likelihood=-70.819097, Pseudo R2=0.3977, Prob.>chi^2^=0.0000

Mini-Mental State Examination (MMSE), Screen for Caregiver Burden (SCB), Neuropsychiatric Inventory (NPI)
